# Association between dietary vitamin D intake and low muscle mass in US adults: results from NHANES 2011–2018

**DOI:** 10.3389/fnut.2024.1471641

**Published:** 2024-10-30

**Authors:** Ye Tong, Yilin Teng, Xiaoming Peng, Bocheng Wan, Shaohui Zong

**Affiliations:** ^1^Department of Spine Osteopathic, The First Affiliated Hospital of Guangxi Medical University, Nanning, China; ^2^Wuming Hospital of Guangxi Medical University, Nanning, China

**Keywords:** NHANES, low muscle mass, vitamin D, dietary vitamin D intake, nutrition

## Abstract

**Purpose:**

To investigate the association between dietary vitamin D intake and low muscle mass (LMM) in a representative adult population, accounting for total energy intake and other potential confounders.

**Materials and methods:**

This cross-sectional study utilized data from the National Health and Nutrition Examination Survey (NHANES) involving 8,443 participants. Dietary vitamin D intake was assessed using 24-h dietary recalls, and LMM was defined based on appendicular lean mass (ALM) adjusted for body mass index (BMI). Multivariable logistic regression models were used to examine the association between quartiles of dietary vitamin D intake and the odds of LMM, adjusting for age, gender, race/ethnicity, BMI, total energy intake, and additional covariates.

**Results:**

In Model 1, after adjusting for age, gender, race/ethnicity, BMI, and poverty-to-income ratio, participants in the highest quartile of vitamin D intake had an odds ratio (OR) of 0.54 (95% CI: 0.37–0.79) compared to the lowest quartile, with a *p* for trend <0.001. In Model 2, after further adjustment for total energy intake and several covariates, the association was attenuated but remained borderline significant (*p* for trend = 0.051). In Model 3, after adjusting for additional health-related factors, the OR for the highest quartile was 0.70 (95% CI: 0.47–1.05), with a significant *p* for trend of 0.029.

**Conclusion:**

This study suggests that higher dietary vitamin D intake may be associated with a reduced risk of LMM. Further longitudinal research is needed to confirm these findings and explore potential interactions between vitamin D and other dietary factors in muscle mass preservation.

## 1 Introduction

Low muscle mass (LMM) is increasingly recognized as a critical component of age-related musculoskeletal decline, contributing to frailty, reduced mobility, and higher risk of morbidity and mortality, particularly in older populations (1, 2). While LMM is traditionally linked with aging, emerging evidence suggests that nutritional factors, including vitamin D intake, may play a key role in its development and progression (3–5).

Vitamin D is well-known for its role in calcium homeostasis and bone health, but its importance for muscle function and mass is gaining attention (6, 7). Mechanistically, vitamin D is thought to influence muscle mass by promoting protein synthesis, enhancing mitochondrial function, and reducing inflammation (8). Observational studies have found associations between low serum vitamin D levels and increased risk of muscle weakness and sarcopenia, a syndrome that includes both muscle mass and function loss (9). However, the role of dietary vitamin D intake, as opposed to serum levels, in mitigating the risk of LMM remains less explored, particularly in population-based studies.

Despite the recognized role of vitamin D in muscle health, there is limited research on the relationship between dietary vitamin D intake and LMM. This study aims to explore the association between dietary vitamin D intake and LMM in a representative adult population. By using data from the National Health and Nutrition Examination Survey (NHANES), we seek to assess whether higher vitamin D intake is associated with lower odds of LMM after adjusting for relevant confounders, such as age, sex, BMI, and total energy intake. Understanding this association may help inform public health strategies aimed at preventing LMM through diet and nutrition interventions.

## 2 Materials and methods

### 2.1 Study population

**Figure 1:**
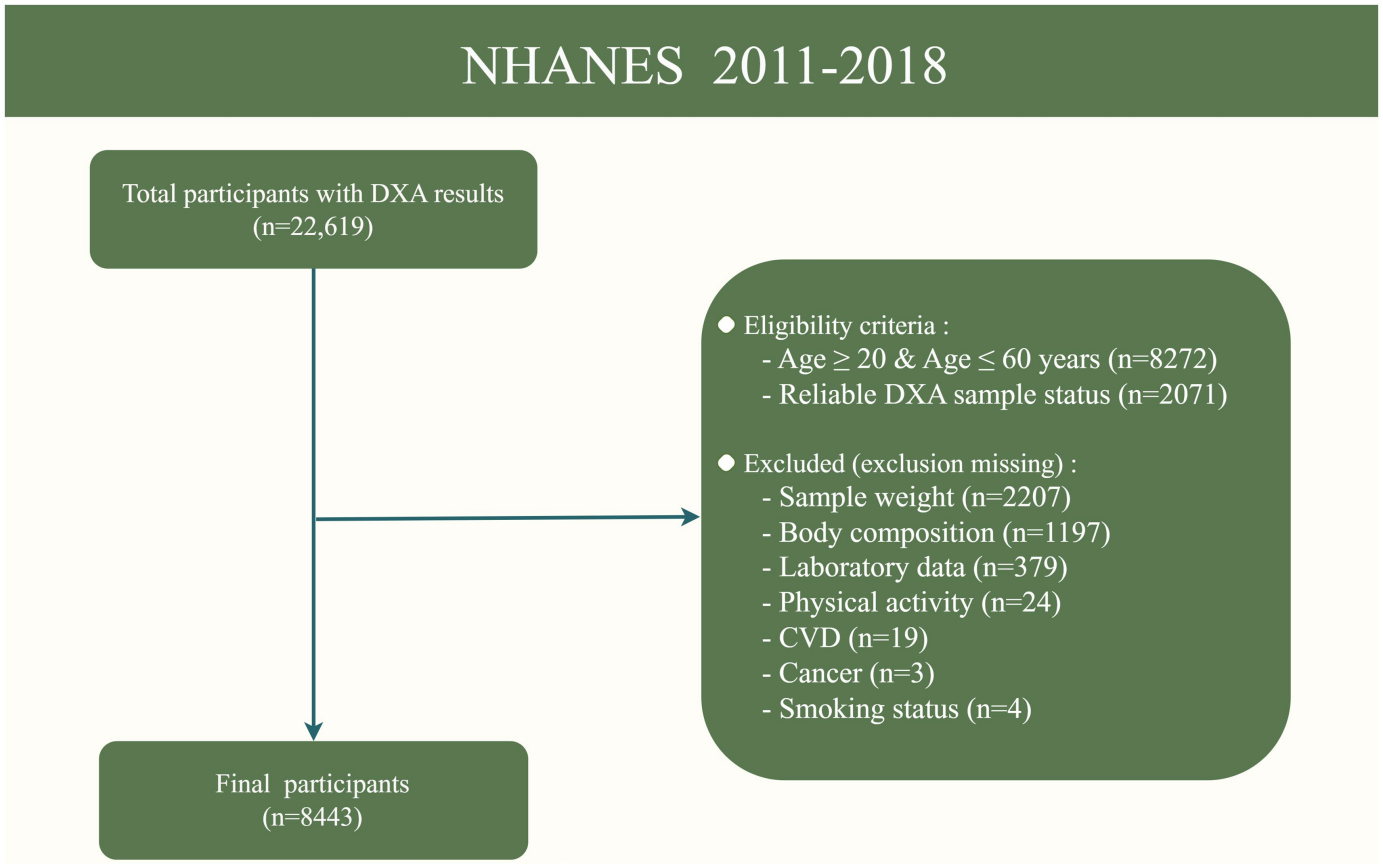
Flowchart of the sample selection from NHANES 2011–2018. DXA, Dual-energy X-ray Absorptiometry; CVD, Cardiovascular disease.

This study was conducted using NHANES data from 2011 to 2018, which included Dual-energy X-ray Absorptiometry (DXA) measures of body composition. NHANES was designed to represent the non-institutionalized U.S. civilian population using a complex, multistage probability sampling method, including oversampling of certain subgroups to produce reliable statistics. All procedural manuals and survey content are indexed and publicly accessible online, ensuring transparency and accessibility for researchers.^1^ For this study, participants aged 20–60 years with complete and reliable DXA measurements were considered eligible for the analysis of LMM. We excluded participants with missing data on key variables, such as sample weight, vitamin D intake, DXA results, BMI, physical activity and other relevant covariates. After applying these criteria, a total of 8,443 participants were included in the final analysis. The participant selection process is illustrated in Figure 1.

### 2.2 Vitamin D intake

Dietary intake interviews were conducted in person at the NHANES mobile examination clinics. Survey participants recalled the specific types and amounts of foods consumed the previous day. Dietary intake was evaluated using two 24-h dietary recalls for each participant. The first 24-h recall was collected in person, and the second was conducted by phone 3 to 10 days later. Vitamin D intake was calculated by averaging the values from the two 24-h recalls. If the second 24-h intake data was missing, the intake from the first 24-h recall was used instead. The dietary intake data were assessed using the automated multiple pass method (10), a structured interview technique consisting of five steps: quick list, forgotten foods, time and occasion, detail cycle, and final probe. After the dietary interview, the caloric and nutrient contents of each reported food and beverage item were coded using the U.S. Department of Agriculture (USDA) Food and Nutrient Database for Dietary Studies (FNDDS).^2^ Information on the consumption of specific food groups was extracted from the Food Patterns Equivalents Database (FPED), with each NHANES data cycle analyzed using its corresponding version of FPED.

### 2.3 Low muscle mass

LMM was defined using the criteria recommended by the Foundation for the National Institutes of Health (FNIH) Sarcopenia Project (11). Specifically, LMM was determined based on ALM adjusted for BMI. Participants were classified as having LMM if their ALM/BMI ratio was less than 0.789 for men and less than 0.512 for women. This definition has been widely used in epidemiological studies and allows for a standardized assessment of muscle mass across different population groups, accounting for variations in body size. The whole-body DXA scans were performed using a Hologic QDR-4500A fan-beam densitometer (Hologic, Inc., Bedford, MA, United States) to assess body composition. Participants were excluded from the DXA examination for several reasons, including pregnancy, self-reported weight over 450 lb. (204 kg), height over 6 ft., 5 in. (195 cm) due to DXA table limitations, and the use of radiographic contrast material (barium) in the past 7 days. Appendicular lean mass (ALM) was calculated as the sum of lean mass for the four limbs.

### 2.4 Covariates

The covariates included in our analysis were age, gender, race/ethnicity (Mexican American, Non-Hispanic White, Non-Hispanic Black, Other race), family poverty-to-income ratio (PIR) (<1, 1–3, >3), body mass index (BMI), total energy intake, diabetes, cardiovascular disease (CVD) status, cancer, smoking status, and drinking status. Physical activity was measured using Metabolic Equivalent (MET) values. Total MET-minutes were estimated by summing the MET-minutes for each activity based on its type and intensity. Participants reporting no regular physical activity in the past 30 days were classified as sedentary. After excluding these sedentary individuals, the remaining participants were divided into gender-specific tertiles according to their physical activity levels, resulting in four categories: sedentary, low, moderate, and high (12). Smoking status was categorized as nonsmoker (having smoked fewer than 100 cigarettes in a lifetime), former smoker (smoked more than 100 cigarettes but not currently smoking), and current smoker (smoked more than 100 cigarettes and currently smoking). Diabetes status was defined using self-reported diagnosis, HbA1c levels ≥6.5%, or fasting plasma glucose (FPG) levels ≥126 mg/dL. Drinking status was classified as never drinker, abstainer, or current drinker based on self-reported alcohol consumption patterns. The presence of CVD and cancer was determined through self-reported physician diagnoses obtained during structured interviews. Furthermore, laboratory measures included serum vitamin D, serum total cholesterol, serum albumin, and serum calcium. Serum vitamin D concentration (Serum 25-hydroxyvitamin D) was classified into four categories: severe deficiency (<25 nmol/L), deficiency (25–49.9 nmol/L), insufficiency (50–74.9 nmol/L), and sufficiency (≥75 nmol/L) (13).

### 2.5 Statistical analysis

Due to the complex sampling design used in NHANES, all analyses were conducted using sample weights. Baseline characteristics were presented unweighted to provide an accurate description of the study cohort. Continuous variables were shown as mean ± standard deviation (SD), while categorical variables were displayed as numbers and percentages (%). The Student’s t-test or Wilcoxon rank-sum test was used to compare continuous variables, and chi-square tests were employed to compare categorical variables. The association between vitamin D intake and sarcopenia was evaluated using multivariable logistic regression (MLR). Vitamin D intake was categorized into quartiles, with the lowest quartile (Q1) serving as the reference group. Model 1 was adjusted for age, gender, race, BMI, and PIR. Model 2 included additional adjustments for total energy intake, serum total cholesterol, serum vitamin D, serum albumin, and serum calcium. Finally, Model 3 was further adjusted for diabetes, CVD, cancer, smoking status, drinking status, and physical activity. To examine the independent effect of vitamin D intake, total energy intake was introduced as a covariate in Models 2 and 3. Before its inclusion, we conducted a Pearson correlation analysis between total energy intake and vitamin D intake, yielding an R value of 0.305 (*p* < 0.001, Supplementary Figure S1), indicating a moderate positive relationship. Additionally, the variance inflation factor (VIF) was calculated as 1.075, demonstrating minimal multicollinearity. Thus, total energy intake was appropriately included in these models to account for potential confounding effects. Results were reported as odds ratios (OR) with 95% confidence intervals (CI). To evaluate the trend between vitamin D intake and LMM, a *p*-value for trend was calculated by treating categorical exposure variables as ordinal in the MLR. All analyses were performed using R software (version 4.4.0), with statistical significance set at *p* < 0.05.

## 3 Results

### 3.1 Baseline characteristics

The baseline characteristics of the study population (*n* = 8,443) are summarized (Table 1), highlighting significant differences between participants with LMM (7.3%) and those without (93%). Participants with LMM were older (43.6 ± 12.0 vs. 39.1 ± 11.8 years, *p* < 0.001), had a higher BMI (35.4 ± 7.6 vs. 28.2 ± 6.2 kg/m^2^, *p* < 0.001), and a greater prevalence of diabetes (21.5% vs. 6.8%, *p* < 0.001) and CVD (9.3% vs. 2.7%, *p* < 0.001). The LMM group also showed a higher proportion of Mexican Americans (26.9% vs. 9.8%, *p* < 0.001). Additionally, participants with LMM had higher total energy intake, lower serum vitamin D, calcium, and albumin levels (all *p* < 0.001), and were more likely to be sedentary (27.5% vs. 14.9%, *p* < 0.001), while current alcohol consumption was lower in this group (59.4% vs. 74.3%, *p* < 0.001). These differences underscore the association between LMM and adverse health and lifestyle factors.

**Table 1:** Baseline characteristics of study participants stratified by the presence or absence of LMM.

		LMM		
Characteristic	Overall, *n* = 8,443 (100%)	None, *n* = 7,712 (93%)	LMM, *n* = 731 (7.3%)	*p*
Age (years)	39.4 ± 11.9	39.1 ± 11.8	43.6 ± 12.0	<0.001
Gender, *n*(%)				0.4
Female	4,387 (50.4%)	4,010 (50.6%)	377 (48.2%)	
Male	4,056 (49.6%)	3,702 (49.4%)	354 (51.8%)	
BMI (kg/m^2^)	28.7 ± 6.6	28.2 ± 6.2	35.4 ± 7.6	<0.001
Race/ethnicity, *n*(%)				<0.001
Mexican American	1,235 (11.1%)	980 (9.8%)	255 (26.9%)	
Non Hispanic Black	1,774 (11.0%)	1,726 (11.6%)	48 (3.3%)	
Non Hispanic White	3,046 (60.6%)	2,847 (61.7%)	199 (46.3%)	
Other race	2,388 (17.3%)	2,159 (16.8%)	229 (23.5%)	
PIR, *n*(%)				<0.001
< 1	1,669 (14.5%)	1,494 (14.1%)	175 (19.4%)	
1–3	3,140 (33.5%)	2,834 (33.0%)	306 (40.5%)	
> 3	3,017 (45.8%)	2,829 (46.9%)	188 (32.1%)	
Unclear	617 (6.2%)	555 (6.1%)	62 (8.0%)	
Total energy intake (kcal/day)				<0.001
Q1	2,111.0 (23.1%)	1,871.0 (22.4%)	240.0 (31.5%)	
Q2	2,111.0 (25.5%)	1,902.0 (25.2%)	209.0 (28.6%)	
Q3	2,111.0 (26.4%)	1,958.0 (26.8%)	153.0 (21.0%)	
Q4	2,110.0 (25.1%)	1,981.0 (25.6%)	129.0 (18.9%)	
Serum total cholesterol (mg/dL)	192.5 ± 40.4	192.0 ± 40.1	198.2 ± 42.7	0.014
Serum calcium (mg/dL)	9.4 ± 0.3	9.4 ± 0.3	9.3 ± 0.3	0.001
Serum albumin (g/dL)	4.3 ± 0.3	4.3 ± 0.3	4.2 ± 0.3	<0.001
Serum Vitamin D (nmol/L)				<0.001
Severe deficiency	399 (3.0%)	357 (2.8%)	42 (5.0%)	
Moderate deficiency	2,611 (23.4%)	2,349 (22.5%)	262 (34.5%)	
Insufficient	3,291 (40.5%)	3,002 (40.6%)	289 (38.8%)	
Sufficient	2,142 (33.1%)	2,004 (34.0%)	138 (21.6%)	
Drinking status, *n*(%)				<0.001
Never drinker	1,758 (16.2%)	1,538 (15.5%)	220 (26.0%)	
Abstainer	680 (7.5%)	590 (7.1%)	90 (12.2%)	
Current drinker	5,684 (73.2%)	5,291 (74.3%)	393 (59.4%)	
Unclear	321 (3.0%)	293 (3.1%)	28 (2.4%)	
Physical activity, *n*(%)				<0.001
Sedentary	1,582 (15.8%)	1,356 (14.9%)	226 (27.5%)	
Low	2,040 (23.6%)	1,863 (23.5%)	177 (25.6%)	
Moderate	2,250 (29.0%)	2,106 (29.6%)	144 (22.0%)	
High	2,571 (31.5%)	2,387 (32.1%)	184 (24.9%)	
Diabetes, *n*(%)				<0.001
No	3,504 (38.4%)	3,246 (38.9%)	258 (31.1%)	
Yes	884 (7.9%)	717 (6.8%)	167 (21.5%)	
Unclear	4,055 (53.8%)	3,749 (54.3%)	306 (47.4%)	
CVD, *n*(%)	326 (3.2%)	265 (2.7%)	61 (9.3%)	<0.001
Cancer, *n*(%)	334 (5.1%)	292 (4.9%)	42 (7.2%)	0.10
Smoking status, *n*(%)				0.2
Nonsmoker	5,196 (59.9%)	4,729 (59.8%)	467 (61.7%)	
Former smoker	1,430 (19.3%)	1,292 (19.2%)	138 (21.0%)	
Current smoker	1,817 (20.8%)	1,691 (21.1%)	126 (17.3%)	
Vitamin D intake (mcg/day)				0.009
Q1	2,111 (24.6%)	1,917 (24.2%)	194 (29.7%)	
Q2	2,111 (25.1%)	1,919 (24.7%)	192 (29.1%)	
Q3	2,111 (25.2%)	1,927 (25.5%)	184 (21.7%)	
Q4	2,110 (25.1%)	1,949 (25.5%)	161 (19.5%)	

LMM, Low muscle mass; BMI, Body mass index; PIR, Poverty-to-Income ratio; CVD, Cardiovascular disease.

### 3.2 Association between vitamin D intake and LMM

**Figure 2:**
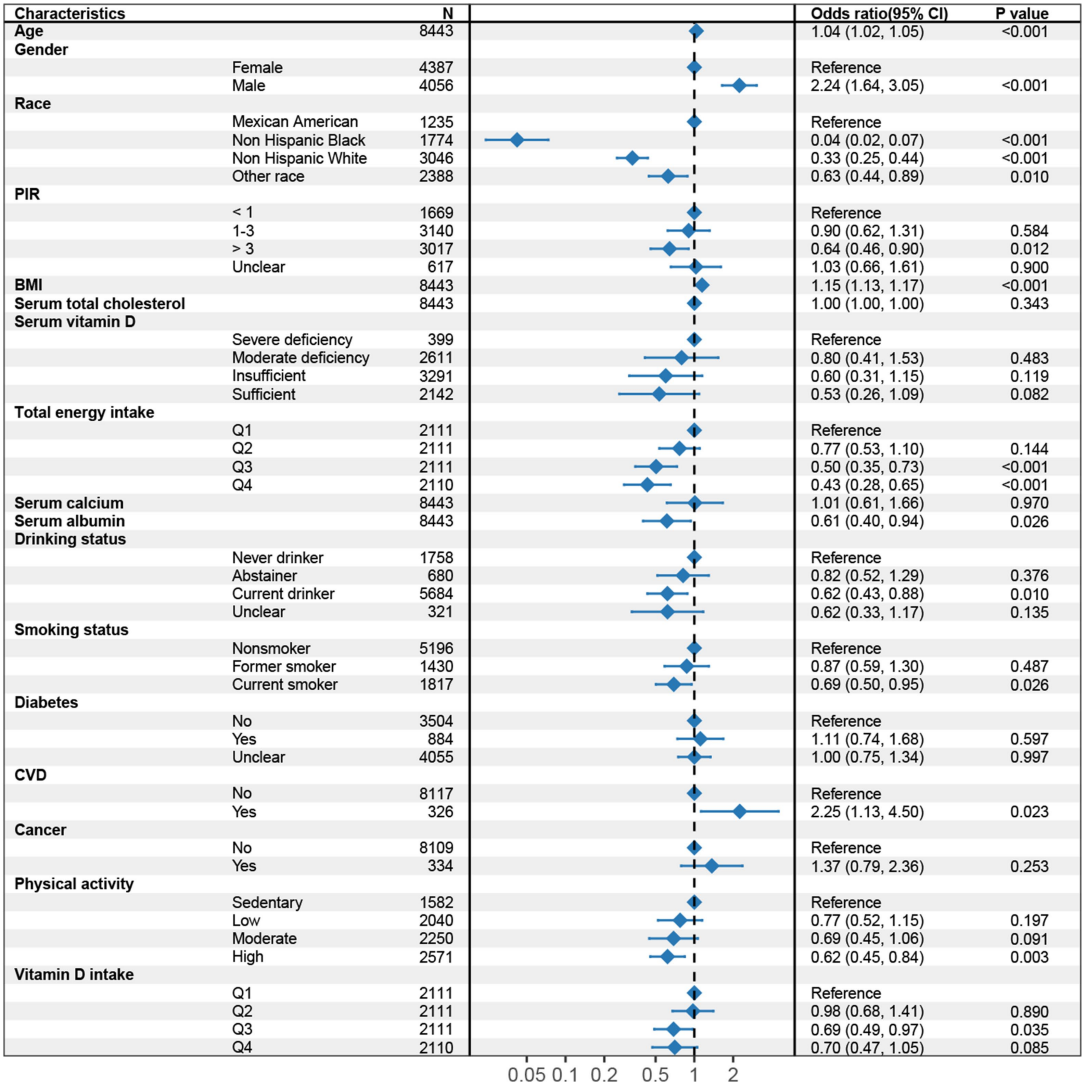
Forest plot of multivariable logistic regression analysis for the association between dietary vitamin D intake and sarcopenia (Model 3, adjusted for age, gender, Race/Ethnicity, BMI, PIR, total energy intake, serum total cholesterol, serum vitamin D, serum albumin, serum calcium, diabetes, CVD, cancer, smoking status, drinking status, and physical activity). CI, Confidence interval; BMI, Body mass index; PIR, Poverty-to-Income ratio; CVD, Cardiovascular disease.

In the multivariable logistic regression models, we assessed the association between dietary vitamin D intake and the risk of LMM, adjusting for various covariates in three models (Table 2). In Model 1 (Supplementary Table S1), participants in the higher quartiles of vitamin D intake (Q3 and Q4) had significantly lower odds of LMM compared to the lowest quartile (Q1), with odds ratios (OR) of 0.57 (95% CI: 0.41–0.79) for Q3 and 0.54 (95% CI: 0.37–0.79) for Q4 (*p* for trend <0.001). In Model 2 (Supplementary Table S2), after adjusting for total energy intake and additional covariates, the association between higher dietary vitamin D intake and lower risk of LMM was attenuated and did not reach statistical significance (Q3: OR = 0.71, 95% CI: 0.50–1.01; Q4: OR = 0.74, 95% CI: 0.50–1.11). The trend across quartiles was borderline significant (*p* for trend = 0.051), suggesting a potential relationship that may warrant further investigation. In Model 3 (Supplementary Table S3; Figure 2), after further adjusting for additional health-related factors, the association between vitamin D intake and LMM reached statistical significance (*p* = 0.044). Participants in Q3 had an OR of 0.69 (95% CI: 0.49–0.97), and those in Q4 had an OR of 0.70 (95% CI: 0.47–1.05), with a significant trend across quartiles (*p* for trend = 0.029).

**Table 2:** Summary of multivariable logistic regression models assessing the association between dietary vitamin D intake and LMM risk.

	OR (95% CI)				*p*	*P* for trend
	Q1	Q2	Q3	Q4		
Model 1	–	0.87 (0.60, 1.26)	0.57 (0.41, 0.79)	0.54 (0.37, 0.79)	<0.001	<0.001
Model 2	–	0.99 (0.69, 1.44)	0.71 (0.50, 1.01)	0.74 (0.50, 1.11)	0.066	0.051
Model 3	–	0.98 (0.68, 1.41)	0.69 (0.49, 0.97)	0.70 (0.47, 1.05)	0.044	0.029

LMM, Low muscle mass; OR, Odds ratio; CI, Confidence interval; CVD, Cardiovascular disease.

Model 1, adjusted for age, gender, Race/Ethnicity, BMI, and PIR; Model 2, adjusted for age, gender, Race/Ethnicity, BMI, PIR, total energy intake, serum total cholesterol, serum vitamin D, serum albumin, and serum calcium; Model 3, adjusted for age, gender, Race/Ethnicity, BMI, PIR, total energy intake, serum total cholesterol, serum vitamin D, serum albumin, serum calcium, diabetes, CVD, cancer, smoking status, drinking status, and physical activity.

## 4 Discussion

This study examined the association between dietary vitamin D intake and low muscle mass (LMM) in a large, representative sample, adjusting for a range of potential confounders. Our findings demonstrate that higher dietary vitamin D intake is associated with a reduced risk of LMM, even after accounting for total energy intake and other relevant covariates. These results align with existing evidence supporting the role of vitamin D in muscle health, but also highlight the complexities of interpreting nutrient-disease relationships, particularly when adjusting for factors such as energy intake.

Vitamin D is known to influence muscle function and structure through several mechanisms. It enhances calcium absorption and influences muscle protein synthesis, both of which are critical for maintaining muscle mass (14). Additionally, vitamin D has been shown to modulate inflammatory pathways, which could further support muscle preservation, particularly in aging populations where inflammation-related muscle degradation is a concern (15). Our findings suggest that dietary vitamin D intake could contribute to the maintenance of muscle mass, consistent with previous studies showing that vitamin D deficiency is associated with muscle weakness and sarcopenia (16–18). While our results indicate an association between higher dietary vitamin D intake and a reduced risk of LMM, these findings should be interpreted with caution. In Model 2, where total energy intake and additional covariates were included, the association was attenuated and did not reach statistical significance (*p* = 0.066). However, the overall trend across quartiles remained borderline significant (*p* for trend = 0.051), suggesting a possible dose–response relationship. In Model 3, after further adjustment for health-related factors, the association regained statistical significance (*p* = 0.044), but the effect size was modest. The overall trend across quartiles remained significant (*p* for trend = 0.029), indicating that higher vitamin D intake may be associated with a lower risk of LMM, though the strength of this association was not robust. The reduction in the strength of the association after adjusting for total energy intake highlights the complexity of disentangling the independent effects of specific nutrients like vitamin D from broader dietary patterns. Total energy intake influences overall dietary patterns, which could confound the relationship between specific nutrients like vitamin D and muscle mass (19). In particular, individuals with higher energy intake may have more diverse diets that include other nutrients beneficial for muscle mass, such as protein and other micronutrients (20). Therefore, while vitamin D may play a role in muscle health, its effect may be part of a more complex, multifactorial dietary environment. Further research, particularly in the form of longitudinal studies or randomized controlled trials, is needed to clarify the role of dietary vitamin D intake in the prevention of LMM and to explore whether certain subgroups may benefit more from increased vitamin D intake.

## 5 Conclusion

In this study, we observed a significant association between higher dietary vitamin D intake and a reduced risk of LMM. These findings add to the growing body of evidence supporting the role of vitamin D in muscle health. However, this association was attenuated after adjusting for total energy intake and other relevant covariates, suggesting that the relationship between vitamin D intake and muscle mass may be influenced by broader dietary patterns or other lifestyle factors. Given the widespread prevalence of vitamin D deficiency and its potential impact on muscle health, increasing dietary vitamin D intake through food fortification or supplementation may represent a valuable public health strategy for preventing muscle mass decline.

## Data Availability

The original contributions presented in the study are included in the article/Supplementary material, further inquiries can be directed to the corresponding author.

## Footnotes

^1^https://www.cdc.gov/nchs/nhanes/index.htm

^2^http://www.ars.usda.gov/nea/bhnrc/fsrg
