# Supplementary material for: Association between dietary vitamin D intake and low muscle mass in US adults: results from NHANES 2011–2018
Source: Front Nutr. 2024 Oct 30;11:1471641. doi: 10.3389/fnut.2024.1471641 (PMC11559427; doi:10.3389/fnut.2024.1471641)

**Supplementary Table 1: Multivariable logistic regression analysis for the association between dietary vitamin D intake and LMM** (Model 1, adjusted for age, gender, Race/Ethnicity, BMI and PIR).

Abbreviations: LMM: Low muscle mass, OR: Odds ratio, CI: Confidence interval, BMI: Body mass index, PIR: Poverty-to-Income ratio.

| **Characteristic** | **OR (95% CI)** | ***p*** |
| --- | --- | --- |
| Age | 1.05 (1.03, 1.06) | <0.001 |
| Gender |  | 0.008 |
| Female | — |  |
| Male | 1.45 (1.09, 1.92) |  |
| Race/ethnicity |  | <0.001 |
| Mexican American | — |  |
| Non Hispanic Black | 0.05 (0.03, 0.08) |  |
| Non Hispanic White | 0.29 (0.21, 0.38) |  |
| Other race | 0.67 (0.47, 0.93) |  |
| PIR |  | <0.001 |
| < 1 | — |  |
| 1-3 | 0.79 (0.55, 1.12) |  |
| > 3 | 0.50 (0.37, 0.69) |  |
| Unclear | 0.91 (0.57, 1.46) |  |
| BMI | 1.16 (1.14, 1.18) | <0.001 |
| Vitamin D intake |  | <0.001 |
| Q1 | — |  |
| Q2 | 0.87 (0.60, 1.26) |  |
| Q3 | 0.57 (0.41, 0.79) |  |
| Q4 | 0.54 (0.37, 0.79) |  |

**Supplementary Table 2: Multivariable logistic regression analysis for the association between dietary vitamin D intake and LMM** (Model 2, adjusted for age, gender, Race/Ethnicity, BMI, PIR, total energy intake, serum total cholesterol, serum vitamin D, serum albumin and serum calcium).

Abbreviations: LMM: Low muscle mass, OR: Odds ratio, CI: Confidence interval, BMI: Body mass index, PIR: Poverty-to-Income ratio.

| **Characteristic** | **OR (95% CI)** | ***p*** |
| --- | --- | --- |
| Age | 1.05 (1.03, 1.06) | <0.001 |
| Gender |  | 0.004 |
| Female | — |  |
| Male | 1.99 (1.50, 2.65) |  |
| Race |  | <0.001 |
| Mexican American | — |  |
| Non Hispanic Black | 0.04 (0.02, 0.07) |  |
| Non Hispanic White | 0.33 (0.24, 0.43) |  |
| Other race | 0.65 (0.47, 0.92) |  |
| PIR |  | 0.007 |
| < 1 | — |  |
| 1-3 | 0.86 (0.60, 1.23) |  |
| > 3 | 0.59 (0.43, 0.81) |  |
| Unclear | 1.03 (0.66, 1.60) |  |
| BMI | 1.15 (1.13, 1.17) | <0.001 |
| Total energy intake |  | <0.001 |
| Q1 | — |  |
| Q2 | 0.74 (0.53, 1.04) |  |
| Q3 | 0.48 (0.34, 0.70) |  |
| Q4 | 0.40 (0.27, 0.59) |  |
| Serum total cholesterol | 1.00 (1.00, 1.00) | 0.5 |
| Serum Vitamin D |  | 0.045 |
| Severe deficiency | — |  |
| Moderate deficiency | 0.74 (0.40, 1.38) |  |
| Insufficient | 0.55 (0.30, 1.01) |  |
| Sufficient | 0.48 (0.24, 0.96) |  |
| Serum calcium | 1.05 (0.64, 1.72) | 0.8 |
| Serum albumin | 0.63 (0.42, 0.94) | 0.019 |
| Vitamin D intake |  | 0.066 |
| Q1 | — |  |
| Q2 | 1.0 (0.69, 1.44) |  |
| Q3 | 0.71 (0.50, 1.01) |  |
| Q4 | 0.74 (0.50, 1.11) |  |

**Supplementary Table 3:** **Multivariable logistic regression analysis for the association between dietary vitamin D intake and LMM** (Model 3, adjusted for age, gender, Race/Ethnicity, BMI, PIR, total energy intake, serum total cholesterol, serum vitamin D, serum albumin, serum calcium, diabetes, CVD, cancer, smoking status, drinking status, and physical activity).

Abbreviations: LMM: Low muscle mass, OR: Odds ratio, CI: Confidence interval, BMI: Body mass index, PIR: Poverty-to-Income ratio, CVD: Cardiovascular disease.

| **Characteristic** | **OR (95% CI)** | ***p*** |
| --- | --- | --- |
| Age | 1.04 (1.02, 1.05) | <0.001 |
| Gender |  | <0.001 |
| Female | — |  |
| Male | 2.24 (1.64, 3.05) |  |
| Race |  | <0.001 |
| Mexican American | — |  |
| Non Hispanic Black | 0.04 (0.02, 0.07) |  |
| Non Hispanic White | 0.33 (0.25, 0.44) |  |
| Other race | 0.63 (0.44, 0.89) |  |
| PIR |  | 0.051 |
| < 1 | — |  |
| 1-3 | 0.90 (0.62, 1.31) |  |
| > 3 | 0.64 (0.46, 0.90) |  |
| Unclear | 1.03 (0.66, 1.61) |  |
| BMI | 1.15 (1.13, 1.17) | <0.001 |
| Total energy intake |  | <0.001 |
| Q1 | — |  |
| Q2 | 0.77 (0.53, 1.10) |  |
| Q3 | 0.50 (0.35, 0.73) |  |
| Q4 | 0.43 (0.28, 0.65) |  |
| Serum total cholesterol | 1.00 (1.00, 1.00) | 0.3 |
| Serum Vitamin D |  | 0.081 |
| Severe deficiency | — |  |
| Moderate deficiency | 0.80 (0.41, 1.53) |  |
| Insufficient | 0.60 (0.31, 1.15) |  |
| Sufficient | 0.53 (0.26, 1.09) |  |
| Serum calcium | 1.01 (0.61, 1.66) | >0.9 |
| Serum albumin | 0.61 (0.40, 0.94) | 0.019 |
| Drinking status |  | 0.026 |
| Never drinker | — |  |
| Abstainer | 0.82 (0.52, 1.29) |  |
| Current drinker | 0.62 (0.43, 0.88) |  |
| Unclear | 0.62 (0.33, 1.17) |  |
| Smoking status |  | 0.060 |
| Nonsmoker | — |  |
| Former smoker | 0.87 (0.59, 1.30) |  |
| Current smoker | 0.69 (0.50, 0.95) |  |
| Diabetes |  | 0.6 |
| No | — |  |
| Yes | 1.11 (0.74, 1.68) |  |
| Unclear | 1.00 (0.75, 1.34) |  |
| CVD |  | 0.017 |
| No | — |  |
| Yes | 2.25 (1.13, 4.50) |  |
| Cancer |  | 0.2 |
| No | — |  |
| Yes | 1.37 (0.79, 2.36) |  |
| Physical activity |  | 0.015 |
| Sedentary | — |  |
| Low | 0.77 (0.52, 1.15) |  |
| Moderate | 0.69 (0.45, 1.06) |  |
| High | 0.62 (0.45, 0.84) |  |
| Vitamin D intake |  | 0.044 |
| Q1 | — |  |
| Q2 | 0.98 (0.68, 1.41) |  |
| Q3 | 0.69 (0.49, 0.97) |  |
| Q4 | 0.70 (0.47, 1.05) |  |

**Supplementary Figure 1**: Correlation between total energy intake and dietary vitamin D intake.


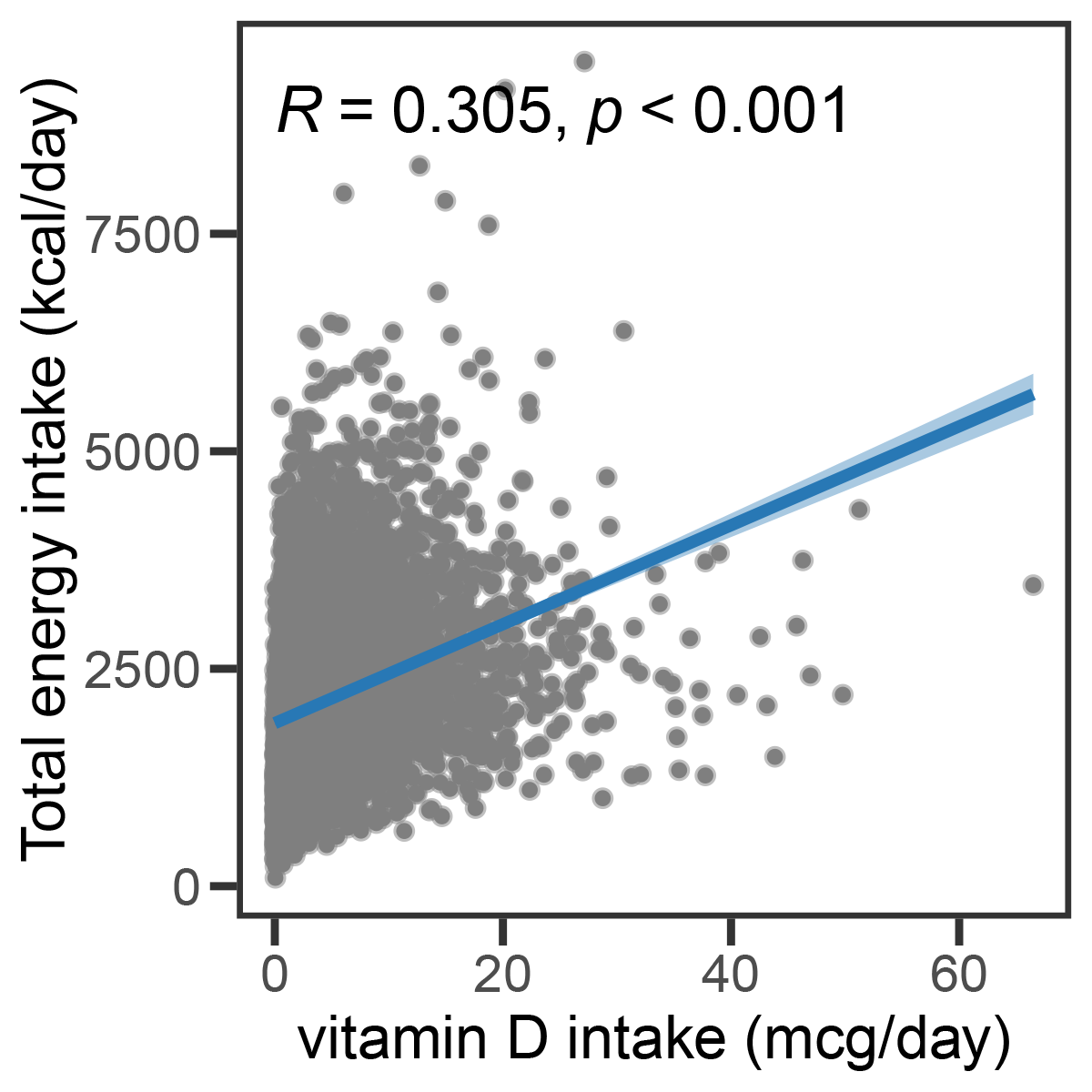

Supplement: Supplementary file 1 [file Data_Sheet_1.docx]
